# Delayed Manifestation of Shunt Nephritis: A Case Report and Review of the Literature

**DOI:** 10.1155/2017/1867349

**Published:** 2017-04-09

**Authors:** Michael Babigumira, Benjamin Huang, Sherry Werner, Wajeh Qunibi

**Affiliations:** ^1^Division of Nephrology, University of Texas Health Science Center at San Antonio, 7703 Floyd Curl Drive, MSC 7882, San Antonio, TX 78229, USA; ^2^San Antonio Uniformed Services Health Education Consortium, San Antonio, TX, USA

## Abstract

We present an unusual case of shunt nephritis in a 39-year-old male who presented 21 years after placement of a ventriculoperitoneal (VP) shunt. He complained of fevers, headaches, dizziness, and urticarial plaques on arms, trunks, and legs and was found to have anemia, low complement levels, elevated serum creatinine, proteinuria, and new onset microhematuria. Blood and urine cultures were negative. Renal biopsy showed features of acute tubulointerstitial nephritis attributed to vancomycin use. Glomeruli showed increased mesangial hypercellularity and segmental endocapillary proliferation. Immunofluorescence showed focal IgM and C3 staining. Electron microscopy revealed small subendothelial electron-dense deposits. Symptoms and renal insufficiency appeared to improve with antibiotic therapy. He was discharged and readmitted 2 months later with similar presentation. CSF grew* Propionibacterium acnes* and shunt hardware grew coagulase-negative* Staphylococcus*. He completed an intravenous antibiotic course and was discharged. On 1-month follow-up, skin lesions persisted but he was otherwise asymptomatic. Follow-up labs showed significant improvement. We did a brief systematic review of the literature on shunt nephritis and report our findings on 79 individual cases. In this review, we comment on the presentation, lab findings, pathological features, and management of this rare, potentially fatal, but curable disease entity.

## 1. Introduction

Hydrocephalus is a pathophysiologic condition due to the accumulation of cerebrospinal fluid (CSF) in the cerebral ventricles [1]. The usual surgical treatment of hydrocephalus is drainage of excess CSF from the brain to another body cavity [shunting]. Historically, the shunts employed were ventriculoatrial (VA), ventriculoperitoneal (VP), and ventriculojugular (VJ) [2, 3], although nonshunt surgical techniques such as endoscopic third ventriculostomy (ETV) are increasingly being accepted [4]. These shunts are associated with a myriad of complications [5, 6] and unique among these is an immunologic phenomenon termed shunt nephritis. This refers to a glomerulonephritis characterized by proteinuria and hematuria which is associated with chronically infected shunts and usually resolves after treatment of the infection, removal of the shunt, or both. Since the description of the first case by Black et al. in 1965 [7], several patients in the literature have been reported to have shunt nephritis. While the overall incidence of shunt infections can range from 7% to 27% [6, 8], the rate of shunt nephritis is considerably much lower with some estimates suggesting its occurrence as low as 0.8% [6].

## 2. Case Report

A 39-year-old male was admitted for work-up of occult infection due to recurrent fevers, nausea, nonbilious emesis, and occipital headache for three weeks. Prior history was notable for traumatic brain injury in 1995 complicated by subarachnoid hemorrhage with subsequent placement of a ventriculoperitoneal (VP) shunt.

At presentation, the patient was tachycardic (HR: 130 per minute) and febrile (103 F) with urticarial plaques involving trunk, arms, and legs. Initial laboratory studies were notable for serum creatinine of 2.0 mg/dL, hemoglobin of 9.7 g/dL, C3 of 45 mg/dL (normal: 98–162), C4 of 9 mg/dL (normal: 16–43), and rheumatoid factor > 100 international units/mL (normal: <15). Urine microscopy did not reveal dysmorphic red cells or cellular casts. Cerebrospinal fluid showed elevated protein, decreased glucose, and gram stain with moderate leukocytosis but no organisms. Brain computed tomography indicated stable findings of his prior surgeries. The patient was given intravenous vancomycin. During the admission, a 24-hour urine collection revealed 1.4 grams of protein. Blood and cerebrospinal cultures remained negative for growth. However, serum creatinine only improved to 1.6 mg/dL (baseline: 0.98–1.1 mg/dL), so a renal biopsy was performed. Following the biopsy, the patient's renal insufficiency began improving, and this recovery was thought to be secondary to successful treatment of the infection. Vancomycin was discontinued at this time in light of negative blood cultures and the consultant neurosurgeon did not feel shunt removal was necessary at that time.

With regard to the renal biopsy, the glomeruli showed mesangial hypercellularity and segmental endocapillary proliferation with a single glomerular crescent containing hyaline droplets. Another glomerulus showed a sclerotic segment. There was no evidence of tubular cell vacuoles. The glomerular basement membranes were not thickened. Immunofluorescence was done with fluorochrome conjugated antibodies against human IgG, IgA, IgM, C3, C1q, fibrin, kappa light chain, and lambda light chain, using appropriate controls. A total of four glomeruli were identified. The glomeruli showed focal segmental 2+ IgM and 3+ C3 mesangial staining in three of the glomeruli. The glomeruli were negative for the other stains. The tubular basement membranes were negative for all of the aforementioned stains.

**Figure 1:**
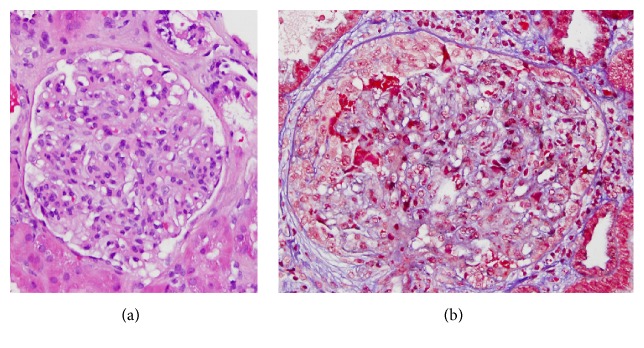
Light microscopy H&E (a) and PAS (b) showing mesangial hypercellularity.

**Figure 2:**
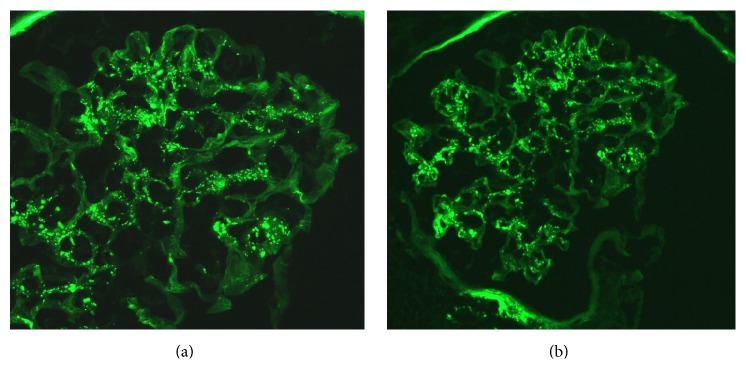
Immunofluorescence positive for C3 (a) and IgM (b) deposition in the mesangium.

**Figure 3:**
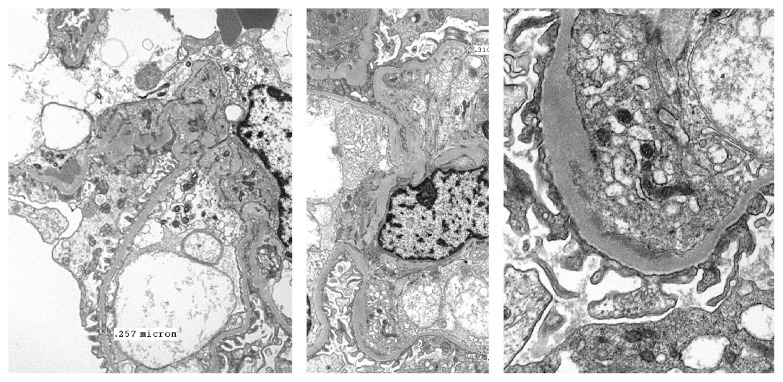
Electron microscopy images showing subendothelial electron-dense deposits.

Electron microscopy showed that the foot processes of the podocytes were subtotally effaced. Rare, small, subendothelial osmiophilic electron-dense deposits were identified. Subepithelial deposits were not present. Spikes or duplicated basement membranes were not identified. Segmentally, there were increased numbers of endothelial and mononuclear cells. The mesangial matrix was mildly expanded and there was slightly increased mesangial cellularity with a few osmiophilic electron-dense deposits identified in the mesangium. These electron-dense deposits were not identified in the tubular basement membranes (see Figures 1–3). The final biopsy report's diagnosis was acute interstitial nephritis with tubular epithelial necrosis and segmental endocapillary proliferation with increased mesangial hypercellularity.

Two months after the renal biopsy, the patient was readmitted for recurrent symptoms similar to his initial presentation. Vital signs and exam were unremarkable. Laboratory data on readmission were notable for serum creatinine of 1.7 mg/dL, hemoglobin of 8.8 g/dL, C3 of 63 mg/dL, C4 of 15 mg/dL, and rheumatoid factor > 100 international units/mL. Cranial imaging did not show acute findings. On subsequent hospital day, the patient had repeat cerebrospinal fluid studies which showed the presence of Gram-positive organisms. He was restarted on antibiotics and the VP shunt was removed emergently. Cerebrospinal fluid culture resulted in the growth of* Propionibacterium acnes* and shunt hardware culture grew coagulase-negative* Staphylococcus*. As per infectious disease consultant's recommendations, the patient was treated with two weeks of intravenous vancomycin and ampicillin. Repeat blood cultures were negative and no further antimicrobial treatment was required.

At 1-month follow-up in the clinic, the patient had persistent skin lesions; he was otherwise asymptomatic with no headaches, dizziness, nausea, vomiting, fevers, or chills. Microscopic hematuria had completely resolved, C3 had increased to near normal levels, and serum creatinine had improved but not to baseline. Mild proteinuria persisted. Rheumatoid factor was normal. Serum creatinine was 1.2 and hemoglobin was 11.2 g/dL.

## 3. Discussion

### 3.1. Methodology

We ran the MeSH terms “shunt” and “nephritis” through 3 medical databases, PUBMED, SCOPUS, and Web of Science, yielding a combined total of 243 search results. After eliminating duplicates, articles not relevant to our search, and those written in a language other than English, we were left with 76 relevant articles that included editorials, case reports, case series, and discussions. We identified 79 individual cases of shunt nephritis from 58 articles in this literature, which we further reviewed [7, 9–65].

### 3.2. Pathogenesis

The pathogenetic mechanism of shunt nephritis is not clear. It is hypothesized that the hydrophobic shunt material, acting as a nidus of infection, coupled with biofilm produced by bacteria promotes bacterial growth and bacteremia [10]. The released bacterial antigen results in formation of circulating immune complexes [66, 67], which are deposited in the glomeruli and activate the complement system [29, 32]. In this regard, Dobrin et al. demonstrated low total hemolytic complement, C1–C7, and elevated C9 in 3 patients with shunt nephritis, suggesting classical pathway activation [68]. While Strife et al. noted similar findings in a series of 4 patients with shunt nephritis, they also observed an association of elevated mixed serum cryoglobulins with the hypocomplementemia [69]. These mixed cryoglobulins decreased with treatment of the nephritis along with resolution of hypocomplementemia, suggesting a role in the pathogenesis of the disease. Although the composition of the serum cryoglobulins was different from that of the glomerular deposits, they posited that deposition of these immune complexes in the glomeruli and inability to penetrate the basement membrane result in subendothelial immune complex accumulation, mesangial proliferation, and mesangial interposition, all frequently encountered on renal pathology [70]. Several authors have also demonstrated bacterial antigens in the glomeruli, further bolstering this hypothesis [13, 14, 25, 45]. On the contrary, Vella et al. propose that the source of antigen is the bioprosthetic shunt material which independently triggers a humoral immune response that results in shunt nephritis [71].

### 3.3. Microbiology

Analysis of microbiology from the 79 patients we reviewed is shown in Table 1. Approximately half of the CSF and shunt cultures were negative, while close to one-third of the blood cultures were negative. Four patients had negative blood, CSF, and shunt cultures, so-called culture-negative shunt nephritis [9, 43, 60, 61]. When cultures were positive, the* coagulase-negative Staphylococcal* (CNS) species (including* Staphylococcus epidermidis* and* Staphylococcus albus*) were predominant, irrespective of the type of culture specimen. These were closely followed by* Propionibacterium acnes (P. acnes)*. One patient had a CSF culture positive for more than one bacterium [72], while another grew different bacteria in blood and CSF [9]. Our patient grew* P. acnes* in CSF and shunt cultures grew* Staphylococcus epidermidis* (mixed infection) which is unusual. It is important to note that, in the past,* P. acnes* and CNS were considered contaminants. But the demonstration of the respective bacterial antigens in the blood and glomeruli of patients with shunt nephritis suggests a pathogenic role in the disease process [11, 32]. Of note also is the fact that it may take up to 14 days to grow* Propionibacterium* in cultures [73] and the diagnosis may be missed by terminating cultures prematurely.

### 3.4. Epidemiology

From the 79 patients with shunt nephritis that we reviewed, there is a slight male predominance, that is, 43 males versus 36 females. Patients' ages at presentation range from 1 year to 74 years, with an average age of 21 years. More than 75% of patients had a VA shunt, with a minority having either a VP or VJ shunt. 10% of patients had their shunt modality revised before onset of nephritis. Shunt characteristics are outlined in Table 2. We defined shunt duration as time from index insertion of shunt to onset of first nephritic symptoms irrespective of shunt revision surgery. We recognize that revision surgeries may influence shunt infections, but this information was not easily obtainable from the literature we reviewed. Our shunt duration definition is a departure from the definition used in a recent review, that is, time from last shunt surgery to onset of clinical signs and symptoms [74]. In our series, average shunt duration was 5.8 years. The range of shunt duration was 1 month to 20 years. Our patient presented 21 years after VP shunt placement, making his case rather unusual.

### 3.5. Clinical Presentation

Shunt nephritis may present with variable nonspecific signs and symptoms, most commonly hematuria, fever, hypertension, and hepatosplenomegaly (see Table 3). Less commonly, shunt nephritis may manifest with skin rashes and arthralgia. One case report noted very unusual and unexplained symptoms of fevers and myalgia only when the patient was taking showers [16]. Anemia is thought to be due to decreased erythropoietin production and/or iron deficiency [50] and hepatosplenomegaly secondary to removal of formed immune complexes by the reticuloendothelial system [75]. Arthritis and arthralgia have been attributed to the formation of circulating immune complexes in the synovium [56]. The pathophysiology of urticaria and skin rashes is less well understood but is thought to be immune-mediated [34]. Common lab abnormalities include proteinuria, anemia, hypocomplementemia, and elevated serum creatinine. Positive serum cryoglobulins and rheumatoid factor are seen infrequently.

Nephrotic range proteinuria can be as massive as 38 g per day [63] and the anemia can be severe with hemoglobin as low as 4 g/dL [55]. Acute renal failure (ARF), defined as significant rise in serum creatinine above baseline or significant decline in estimated glomerular filtration rate (GFR), was reported in 46 patients. However, unlike serum creatinine, ARF was not consistently or uniformly reported in all the literatures reviewed. We therefore opted to use serum creatinine of greater than or equal to 1 (arbitrary value) as an alternative marker of renal dysfunction, even though it is inherently less accurate than the ARF definition cited above. Using this measure, over half of the patients reviewed had abnormal renal function (see Table 4). We do recognize the limitations of using serum creatinine as a gauge of renal function since it does not account for ARF in patients with baseline chronic kidney disease. But since no other measure of renal function was consistently reported in all of the reviewed cases, we settled for serum creatinine as a compromise. Some patients do present with positive anti-neutrophil cytoplasmic antibody (ANCA) titers, all antiproteinase 3 positive, with decrease in titers after treatment of the shunt nephritis [15, 31, 36, 42]. The exact mechanism of ANCA production is unclear [31]. The low complements, as discussed previously, may represent activation of the classical pathway as the disease unfolds [69]. For disease screening and diagnostic purposes, Bayston et al. have proposed the use of Anti Staphylococcus Epidermidis Titer (ASET) after they demonstrated that ASET rises predictably in patients with VA shunts colonized by* Staphylococcus epidermidis* [61, 76, 77]. While the ASET is a welcome addition to the diagnostic armamentarium, no single symptom, sign, or lab finding should be considered in isolation when shunt nephritis is suspected. Wyatt et al. suggest that complement levels be monitored until normalization and only be repeated when a relapse of nephritis is suspected [62]. Our patient presented with fever, anemia, microscopic hematuria, proteinuria, hypocomplementemia, renal insufficiency, positive rheumatoid factor, and urticarial plaques, all consistent with features of shunt nephritis described above.

### 3.6. Pathology

Of the 79 shunt nephritis patients reviewed, 62 had renal biopsies with 8 of these patients having repeat biopsies after treatment of the nephritis. The majority of these biopsies reveal a mesangial proliferative and membranoproliferative (MPGN) pattern of glomerulonephritis on light microscopy (see Table 5).

Immunofluorescence was done in approximately two-thirds of the renal biopsies with mesangial IgM, C1q, and C3 deposition being the most common finding. Relative intensities of immunoglobulin deposition were inconsistently reported, so we excluded them from our analysis (see Table 6). Out of the 62 biopsies reported in this literature review, electron microscopy was performed in only 28 instances, of which 18 (64%) had subendothelial deposits and 11 (39%) had mesangial deposits. Only 1 biopsy reported subepithelial deposits [53]. This further confirms the immune complex nature of the disease. Six biopsies had crescents [9, 19, 21, 53, 59, 64]. All the patients that had repeat biopsies after treatment of shunt nephritis demonstrated improvement in histologic findings [24, 28, 59, 60, 62, 63, 65]. One renal biopsy was initially read as amyloidosis but later retrospectively changed to “shunt nephritis” after signs and symptoms resolved with therapy [78].

Our patient's biopsy report showed features of tubulointerstitial nephritis that was attributed to vancomycin use. The glomeruli showed increased mesangial hypercellularity and segmental endocapillary proliferation, while immunofluorescence was positive for IgM and C3. Electron microscopy showed subendothelial deposits (see Figure 3).

### 3.7. Treatment

Therapy for this unique form of glomerulonephritis is multifaceted, including medical (antibiotics) and surgical approaches (shunt removal). Even though some cases of shunt nephritis show improvement with antibiotics alone [13, 14, 37, 40, 44, 54], best results are achieved with removal of the infected shunt, along with a course of IV antibiotics to clear the infection. If hydrocephalus remains a clinical concern, a temporary external ventricular drain may be placed. Some authors advocate administration of intraventricular antibiotics during this period [74]. Once the infection is resolved, it may be prudent to consider nonshunt surgical techniques such as ventriculocisternostomy for persistent hydrocephalus [16]. If a shunt is still necessary, VP shunts appear to be more preferable to VA or VJ shunts (see Table 7). The use of antibiotic-coated shunts and perioperative antibiotic prophylaxis may play a role in the prevention of shunt infection [79, 80].

For the purposes of this review, we defined full renal recovery as complete resolution of hematuria, proteinuria, and renal dysfunction, while partial renal recovery was defined as resolution of one or more of hematuria, proteinuria, or renal dysfunction but not all. As described earlier, renal dysfunction was arbitrarily defined as serum creatinine greater than or equal to 1. The average time to achieve renal recovery (complete or partial resolution of hematuria, proteinuria, and renal dysfunction) is 9.5 months. As illustrated in Table 8, the majority of patients with renal recovery had the shunt removed. From our analysis, it appears that patients with full renal recovery had shorter average duration of shunt placement as compared to the ones with partial renal recovery and those that progressed to ESRD. This may suggest that longer residence of an infected shunt correlates with worse renal outcomes. While concurrent antibiotic therapy may play an additional role in renal recovery, only a small minority of patients in this review did not receive any antibiotics (see Table 9). Our brief analysis did not account for other factors, such as age or significant comorbidities, which may influence renal recovery in shunt nephritis.

Of the 6 deaths that were reported, only 1 was conclusively attributed to renal failure [40], with the rest being unknown or secondary to surgical complications of hydrocephalus [7, 32, 49, 64]. It appears that immune suppression therapy does not attenuate this disease process and may even contribute to negative outcomes [31, 32, 34, 64]. Indeed, one patient got steroids for 2 years followed by cyclophosphamide for presumed ANCA disease, but nephritis resolved only after removal of the shunt [15]. An unusual case of kidney donation by a deceased patient with shunt nephritis described remarkable resolution of renal disease in the recipient [26]; this observation further supports the notion that removal of the injurious immunologic environment is a means of cure. Our patient had the shunt in place for 21 years; the infection was initially treated with antibiotics, but meaningful symptomatic and renal recovery was not achieved until the shunt was removed. In hindsight, our patient's initial presumed diagnosis of acute interstitial nephritis attributed to vancomycin therapy was flawed and should have been revised to “shunt nephritis.”

## 4. Conclusion

Shunt nephritis may manifest with many nonspecific symptoms, signs, and lab findings. If not quickly recognized, diagnostic delay may lead to irreversible chronic renal disease and possibly death. So nephrologists should maintain a high degree of suspicion in patients who present with indwelling CSF shunts and renal disease suggestive of nephritis.

## Figures and Tables

**Table 1:** Microbiologic characteristics of shunt nephritis.

Microorganism	Blood cultures (%)	CSF cultures (%)	Shunt cultures (%)
Negative^*∗*^	27	56	53
*C. bovis*	3	1	0
*M. bovis*	1	1	0
*Diphtheroids*	3	3	1
*Micrococcus*	3	1	4
*G. morbillorum*	1	1	1
*P. aeruginosa*	1	0	4
*S. viridans*	1	0	0
*P. acnes*	10	6	10
*Staphylococcus epidermidis*	19	11	9
*Staphylococcus albus*	15	9	11
*Coagulase-negative Staphylococcus *	13	8	5
*Proteus*	1	0	0
*Staphylococcus aureus*	3	1	1
Mixed infection	0	1	0

*Total*	*100*	*100*	*100*

^*∗*^Culture results reported as negative or not reported at all.

**Table 2:** Characteristics of shunts in shunt nephritis.

Type of Shunt	%
Ventriculoatrial (VA)	76
Ventriculojugular (VJ)	9
Ventriculoperitoneal (VP)	5
Revisions, VP/VA	10

*Total*	*100*

**Table 3:** Presenting signs and symptoms of shunt nephritis.

Clinical finding	%
Hematuria	87
Fever	67
Hypertension	35
Hepatosplenomegaly	23
Edema	15
Skin rash	8
Splenomegaly	6
Arthralgia	4
Altered mental status	4
Seizure	4
Failure to thrive	4

**Table 4:** Lab findings in shunt nephritis.

Lab finding		Number
Proteinuria	Median, 19.4 g (range: 0.5–38)	68
Anemia	Avg., 7.8 g/dL (range: 4–11.5)	58
Serum complements	Low	57
Serum creatinine	Greater than 1/abnormal	43
Acute renal failure	Significant rise in serum creatinine above baseline or decline in estimated GFR	46
Serum cryoglobulins	Positive	7
Serum ANCA	Positive	4
Serum RF	Positive	4

**Table 5:** Light microscopy renal biopsy findings.

Biopsy findings	%
Mesangial proliferative GN	52
MPGN	45
Amyloidosis	2
End-stage kidney	2

*Total*	*100*

**Table 6:** Renal biopsy immunofluorescence findings.

Immunofluorescence	% (out of 42)
IgM	83
C3	71
IgG	57
C1q	83
IGA	26
C4	24
Lambda	5
Positive complement	5

**Table 7:** Renal outcomes after shunt nephritis treatment.

	Full renal recovery	Partial renal recovery	Progressed to ESRD	Death
Shunt removed	16	19	3	2
Shunt replaced	5	6	1	1
Converted to VP shunt	7	9	0	0
Antibiotics alone	0	6	0	3

*Total* ^*∗*^	*29*	*40*	*4*	*6*

Full renal recovery: complete resolution of hematuria, proteinuria, and renal dysfunction. Partial renal recovery: resolution of 1 or more of hematuria, proteinuria, and renal dysfunction but not all.

^*∗*^Case of deceased kidney donor with shunt nephritis is excluded.

**Table 8:** Time to renal recovery after shunt nephritis treatment versus average shunt duration.

	%	Time to renal recovery in months (range)	Average shunt duration in years (range)
Death	8	—	4.79 (3.5–15)
Progressed to ESRD	5	—	8.75 (4–14)
Full renal recovery	37	10.8 (1–48)	4.94 (0.25–19)
Partial renal recovery	51	8.4 (0.25–72)	6.28. (0.1–20)

*Total*	*100*	*9.5 (0.25–72)*	*5.8 (0.1*–*20)*

Full renal recovery: complete resolution of hematuria, proteinuria, and renal dysfunction. Partial renal recovery: resolution of 1 or more of hematuria, proteinuria, and renal dysfunction but not all.

**Table 9:** Proportion of patients that received concurrent antibiotic therapy.

	Full renal recovery	Partial renal recovery	Progressed to ESRD	Death
Shunt removed	81%	84%	100%	100%
Shunt replaced	80%	100%	100%	100%
Converted to VP shunt	71%	100%	—	—
